# The British Medical Association

**Published:** 1880-10

**Authors:** 


### Reports of Societies.

### THE BRITISH MEDICAL ASSOCIATION.

Held at Cambridge, En.land, August 10, 11, 12, and 13, 1880.

FIRST DAY—AUGUST 10TH—GENERAL SESSION.

The meeting of the Association opened with a larger attendance than ever before. The first address was that of the President, Dr. Humphreys, and it was pronounced the chief event of the session. The first part of it was devoted to showing how little the University of Cambridge had done for medicine in the past, and what promise of work it gave for the future. Cambridge now has unequalled advantages for teaching anatomy, physiology, and chemistry. Good practical instruction in medicine and surgery is also given. It at present has over one hundred medical students. There is every promise that the University will continue to do still more for medicine.

Dr. Humphreys then spoke of the work of "cumulative observation," which it was the duty of the members of the Association to pursue more vigorously and satisfactorily. "This work," he said, "the work of collective action, the pull-together of eight thousand members of the profession, had been too little attempted, or if attempted, had been productive of too little result. It is, perhaps, the only work in which all, or a large part of the members, can really and fully appreciate, and to which each can contribute his mite. It is almost the only way in which questions relating to the effect of temperamental, climacteric and topographical agencies upon disease, and many others, can be fully investigated and solved. To engage members of the Association as participators in any dicision of such work, would prove one of the most powerful inducements to the cultivation of observation and thought respecting the mass of facts which are passing, now too often unheeded or unnoticed, before their eyes." In order to carry out such a project as this, there would have to be a system adopted. There should be, in the first place, a well-paid secretary or registrar, who would collect, codify, and act with a medical investigation committee. The art of composite photography would be a valuable adjunct whereby typical features of certain conformations, and of the features of temperament and character could be obtained.

At the close of the address it was voted that the Committee of Council be directed to consider how the President's suggestions could be best carried out to a practical result.

Dr. Alfred Carpenter read the annual Report of the Council.

This showed that during the past year the receipts of the Association had been $75,000, and the expenses $60,000· The balance for the year in the treasury is over $10,000. The total membership of the Association at date was 8,052, an increase of about 900 during the year.

The medal of the Association for distinguished services has been given to Dr. William Farr, C D., F.R.S.

The organization of three new branches in Australia was announced.

During the year the Parliamentary Bills Committee had succeeded in getting a new warrant which greatly improved the condition of the army medical service. It had been trying to do a like work for the navy. The same committee had done much to secure the compulsory vaccination of children and to introduce the use of calf's lymph.

The Committees on the Habitual Drunkard's Act, on Hospital Out-patient Reform, and on State Medicine, had no special progress to report.

The registration of infectious diseases had been introduced into thirteen towns and cities; it would doubtless be still further extended in the coming year.

The report of the Committee of Council was adopted.

### Second Day—August 11th—General Meeting.

Dr. John Buckley Bradbury, M.D., F.R.C.P., delivered the address in medicine, choosing for his subject "Modern Scientific Medicine."

Dr. Bradbury made it the object of his address to show what modern medicine now owed to physics and the exact sciences. These were, he said, being introduced more and more into everyday practice, and were slowly but surely tending to take away from medicine the reproach that it was not a science. He gave a brief review of the discoveries and practical uses of the various instruments of precision now employed by specialtists and general practitioners. Dr. Bradbury did not make his review a complete one, but referred chiefly to those instruments which he himself employed in his own work.

By the aid of the *microscope*, an advance, he said, had been made in the diagnosis of idiopathic pernicious anæmia. By it Wilks had discovered that there was in this disease no increase in the colorless blood-globules. This fact, combined with others which the microscope had first brought to light, made it possible to differentiate between anæmia, chlorosis, leukæmia, etc. The vast progress in pathology which the microscope had rendered possible was only referred to. The discovery of the disease trichinosis by Zenker, in 1860, with all its subsequent good results in the saving of human life, was due to the microscope. In view of the recent discoveries by Mr. Powell of a parasite resembling trichinæ in the voluntary muscles of typhoid fever patients, the possibility that that disease is a form of trichinosis was hinted at.

The *thermometer* had done even more than the microscope to place medicine on a scentific basis. The "Treaties on Medical Thermometry," by Professor Wunderlich, had done more than any other work to further the progress of scientific medicine in the last ten years. Owing to it we are able to diagnosticate diseases which before, at a early stage, were confounded as tuberculosis and typhoid fever. We can make more confident prognoses, and use our drugs with more precision. It has led to the antipyretic treatment of fevers, which Dr. Bradbury endorsed. The special value of local themometry was described, and M. Peter's investigations into the parental temperament in pleurisy were given. These show that we can tell by the thermomoter whether the effused fluid is being absorbed or is increasing, and at what rate. The observations of M. Peter in cases of tuberculosis, confirmed by M. Vidal, show that as soon as tubercles occur at any point in the lung, the temperature rises there, and in doubtful cases of phthisis where the physical signs are not developed, local thermometry may assist in arriving at a correct conclusion. M. Peter's investigations in regard to the differences between the local temperatures of the abdominal parietes in ascites and in the various forms of peritonitis were also noticed. The value of temperature observations in localizing lesions of the brain was guardedly endorsed. General thermometry is useful in diagnosing cerebral hemorrhage from alcoholic poisoning; true apoplexy from the apoplectiform seizures of general paresis, disseminated sclerosis, cerebral softening, and uræmic coma.

The *ophthalmoscope* has furnished invaluable means not only of diagnosing diseases of the eye, of helping the recognition of intracranial tumors, syphilis, chronic Bright's disease, acute tuberculosis, tubercular meningitis, cerebral embolism, and locomotor ataxia. Cases of apparent brain disease, but in which the symptoms are really due to anomalies of refraction, recognizable by the microscope, also prove the value of the instrument in general medicine. Illustrations of the use of the instrument in detecting the various diseases above mentioned were given. The ophthalmoscope, Dr. Bradbury concluded, must no longer be regarded as an instrument of use only to the ophthalmic surgeon.

The *laryngoscope* had not materially extended its field during the last ten years. Dr. Marcet had, however, shown that with it laryngeal phthisis could be very easily detected, even before the lungs were affected. The use of the instrument in diagnosing syphilitic laryngitis and in determining the presence, extent, and character of the membrane in diphtheria, was also a recent addition to medicine.

The *sphygmograph* was considered at great length. Dr. Bradbury believed it would prove of great service sometimes in the early diagnosis of Bright's disease. The increase of vascular tension, indicating an arterio-capillary, fibrosis, might precede any structural change in the kidneys; and if detected thus early, the threatened Bright's disease might be kept off The discovery of high arterial tension might be a help in indicating other pathological conditions and in pointing the way to successful treatment. As one of the remedies for high tension, Dr. Bradbury recommended bleeding. He expressed his confidence that bleeding was now a much neglected measure, and that it would be oftener employed in the future. Dr. Bradbury discussed the causation of albuminuria. He also spoke of the way in which Dr. Brunton, through the help of the sphymograph, discovered the value of the nitrate of amyl in angina pectoris.

The use of the aspirator was not dwelt upon at any great length. The speaker referred to his success with it in tapping hydatids of the liver.

The *stethometer* was also passed over briefly. It will sometimes indicate traces of lung disease that no physical examination of other kinds could display. Its chief value is in estimating the probable course of cases of phthisis and pleurisy.

The *spectroscope* is said by Dr. McMunn to enable one to distinguish blood from the bladder or urethra from blood from the kidney; and blood from the stomach from blood from the lungs.

*Electricity* is an agent that has helped both the diagnosis and treatment of disease. By it we are enabled to distinguish between spinal and cerebral lesions in cases of paralyses, and between peripheral and central nervous lesions.

*Metallo and magneto-therapy* were reviewed by the speaker, who regarded the phenomena reported by Charcot and others as deserving more credence than is given by some. The theory of "expectant attention" he did not think sufficient to explain everything regarding the phenomena of transferred sensation.

### Third Day—August 12—General Session.

The members of the Committee of Council for the ensuing year were elected. Mr. Waters read the report of the Medical Reform Committee, which was afterwards discussed. The address in surgery was read by the Chairman of the

Section, Mr. Timothy Holmes, M.A., F.R.C.S. Its subject was "Fergusson and conservative surgery—excision of the knee and hip." Mr. Holmes said that British surgery was a thing to be proud of. English surgeons had particularly distinguished themselves in the practical part—that of healing their patients. Almost all the improvements in operative surgery were of English origin. The orator then paid a tribute to Sir Wm. Ferguson as the father of conservative surgery. In lieu of amputation or the slow decay of chronic disease, he had introduced two almost forgotten operations—excision of the hip and excision of the knee.

Mr. Holmes proceeded then to estimate the present position and value of these operations. He spoke in the deepest scorn of statistical methods, believing they had done more harm than good. By numerical statements, he said, it might be proved that "the homœopathist who soothes the sufferings of dyspeptic gluttons or hysterical fine ladies, are better practitioners than those who are consulted in all serious emergencies."

Dr. Holmes nevertheless introduced some figures showing the prevalence of the practice of incision in some of the great London and provincial hospitals. At Guy's Hospital, the operation for excisiion of the knee was performed eighty-nine times during the five years ending 1878; and at St. Thomas's sixty-two times during the same period. In the large hospitals where excision is performed, he believed that there was an increasing tendency to use it less as an operation of urgency than of expediency, and rather for the purpcse of superseding the expectant treatment than as a substitute for amputation. The tables of Mr. Holmes also showed that there has been a reduction in the mortality of the operation from about twenty-four to less than ten per cent. Though very favorable results were secured by doing this operation under Lister, these results were no better than surgeons had obtained who did not employ this method, but were careful to dress the cases themselves and to leave the parts long undisturbed. Mr. Holmes gave as indications for excision: the strumous cases where the bones are superficially ulcerated; cases in which the synovial membrane has degenerated; cases of limited inflammation tending to necrosis; cases of abscess of the bone not extending too far into the joint; and cases of rheumatoid arthritis. Here, if the patient is healthy and especially if a child, the operation may be tried with fair prospect of success.

As a fair summary of the most recent experience the following propositions were laid down :

1. Excision of the knee is one of the indispensable resources of surgery, and is useful in all three classes of cases, viz., in those where, otherwise, amputation would be indicated; in those where the expectant treatment might succeed, but is dubious; and in cases of vicious anchylosis.

2. As a substitute for amputation, it is indicated in early life, and in non-tuberculous subjects; in cases of limited caries of the bones, of degeneration of synovial membrane, and in some conditions of necrosis of the articular surfaces; possibly also in abscess in the ends of the bones.

3. As a substitute for the expected treatment, it seems to be justified, and is extensively used in cases where the patient's circumstances and the slow progress of the case, render the surgeon hopeless or very doubtful of recovery with sound anchylosis.

4. It is also frequently used, and very successfully, in cases of vicious or deformed anchylosis.

5. Attempts have been made to limit the place of excision by opening the joint and drainage, and by some other partial methods. These attempts have been fairly successful, especially in cases where the affection is rather of the synovial membrane than of the bones, and they deserve more extensive trial than they seem as yet to have obtained.

6. At the same time, the mortality from excision of the knee seems of late years to have been so greatly diminished as to encourage the hope that the limit of age which it has been found necessary hitherto to observe may be extended, and it may be judged prudent to apply the operation to the treatment of the more chronic affections of later life, such as chronic rheumatic arthritis, more extensively than has been done up to the present time.

In cases of hip disease, Mr. Holmes sums up his views as follows: Excision of the hip ought to be very rarely indeed required if the disease be treated properly at its commencement; in cases seen at an advanced stage of the disease, it is chiefly when sequestra exist that the operation is necessary, though it may be advisable as a means of shortening the treatment in other cases also, when the patient cannot obtain the prolonged surgical care essential to natural recovery.

The speaker, in conclusion, showed the evils to the poorer class of the present out-patient hospital system, in that it rendered the treatment of chronic diseases so unsatisfactory. He referred to the scheme now under consideration of establishing provident dispensaries throughout the metropolis of London.

At the close of the address, a resolution of thanks was moved by Sir Henry Thompson, who made a brief and eloquent speech.

The report of the Scientific Grants Committee was then made. [A summary of this has appeared in the RECORD.]

The members of the Joint Committee on State Medicine were re-elected for another year.

The ceremony of the presentation of the Gold Medal of the Association, to Dr. Farr, then took place.

FOURTH DAY—AUGUST 13TH—GENERAL MEETING.

The meeting opened with the address in physiology by Michael Foster, M.D., F.R.S., on the "Relations of Physiology and Pathology—the Professional Aspect of Physiology."

Physiology might be defined, he said, as the actions and reactions of human beings; differing, therefore, from morphology, which treats of the characteristics of form. These two branches of science were converging, and would, in times not far distant, unite; that is to say, we shall then know function by studying structure, and we shall know what is the structure of a living being when we know its function. Dr. Foster showed that the physiological inquiry now goes deeper than the investigation of function simply; it studies the molecular changes of the living cells, the ultimate vital phenomena. This was a fact which proved that physiology is closely related to pathology, that the two sciencies are in fact one, and can no more justly be separated than can the science of meteorology be divided into a science of good weather and a science of bad. The speaker lamented the neglected condition of pathological study in England. There is only one solitary institute in that country devoted to pathological inquiry, and that one exists under difficulties, and occupies only a part of the time of a largely engaged practicing physician. A new chair of pathology, very fully equipped, is however, being erected at Cambridge.

Pathology, the lecturer thought, was not only the rational basis of the healing art, but also an important intellectual equipment for every practitioner who is not simply a machine for prescribing drugs in a dull, mechani cal manner. But the salutary use of pathological doctrines requires a certain critical power, and the building up of this power is one of the chief functions, especially for the active practitioner, of physiological study. Physiology might be considered the watch-dog against vagrant pathological theories. The study of physiology was not only practically useful, but it disciplined the mind to habits of careful reasoning and close observation. This was the case at least in schools where the science is taught as it should be. It was to be regretted that in so many of the metropolitan medical schools physiology was taught by those who gave themselves but partly to the study, most of their time being devoted to a medical or surgical practice.

Dr. Foster compared the two studies, anatomy and physiology. The former was, he believed, most useful as a means of mental discipline, it practical value was small apart from this. In former times it had been advisable to insist rigorously upon this study to the exclusion of physiology. Now, he thought, equal discipline could be obtained from the study of physiology, and, in addition, it would train the mind to better habits and fill it with more useful facts. He would have anatomy hereafter made secondary to physiology.

Following Dr. Foster's address were a number of reports of special committees. That of the Committee on Outpatient Reform was made by its chairman, Mr. Timothy Holmes. He said that the work of the committee was now at a stand still, because it was divided against itself. He believed that nothing could be done to reform out-patient department abuses until some system took its place. The only possible system was that of provident dispensaries. They were now trying to secure the co-operation of the friendly societies so as to make such dispensaries possible.

Votes of thanks were given to the various officers and local authorities.

Of the events outside the general and sectional meetings, one of the most notable was the "Conferring of the Honorary Degrees" upon a large number of eminent physicians and surgeons. The ceremony took place in the Senate House. All the doctors, actual and designate, marched in, wearing scarlet hoods. The undergraduates' gallery was filled with students who made the usual demonstrations. The public orator delivered the degrees to the twelve candidates, accompanying each with a short speech in Latin. Sir Wm. Jenner was introduced as celebrated for his great knowledge and success in the treatment of fever, "quæ pulsat pede æquo pauperum tabernas regumque terras." Referring to Dr. S. D. Gross, he said: "Transfluctus Atlanticos, trans oceanum non jam ut antea 'dissociabilem,' partiæ nostræ ad portus nuper advectus est vir venerabilis quem inter fratres nostros transatlanticos scientiæ chirurgicæ quasi alterum Nestorem nominare ausim." When Sir Wm. Gull's name was mentioned, there was a storm of hoots and hisses from the students which stopped the orator's remarks for some time.

*The Section of Surgery.*—The Section of Surgery was opened with an address by its President, Mr. Wm. S. Savory, F.R.S. It was characterized as "clear, eloquent, and useful rather than calling attention to points already known, but apt to be overlooked, than in recording anything new." The subject was "Constitutional Disturbance."

The term "constitutional disturbance" was, the speaker thought, a good one in its application to the effect of local disease or injury. Such disturbance, it was now known, might be brought about by different agencies; chief among them being the nervous system and the blood. The object of the lecturer was to show that two forms of constitutional disturbance from these two causes existed, and that it was very important to distinguish each of them. Mr. Savory then discussed at some length the physiology of the correlation or "sympathy' between different parts and organs of the body. He showed the delicacy and universality of these sympathies, and the distinction between them and reflex action. Continuing, he said, "But impressive as the evidence is of this natural dependence of parts through the nervous system, it is, nevertheless, clear, that this is not the only agent of sympathy; there is another even more universal, and perhaps more subtile, if in health, more obscure in its operation—the blood. The blood is thus the medium of communicatoin between all parts by virtue of the incessant changes which go on every where between the blood and tissues in nutrition. You remember the aphorism: 'Each single part of the body, in respect to its nutrition, stands to the whole body in relation of a secreted substance,' and Paget's brilliant use of it. And it is not hard to understand that if any part fails to withdraw from the blood, or supply to the blood its proper materials, the blood will be affected, and through it remote parts or organs of the body. The blood may be affected by the introduction of poisons from without, as in cases of wounds, or of morbid changes deep in the tissues; but it may also be affected by local morbid actions and by various forms of perverted nutrition." And the lecturer asserted that between the cases of fever induced by the latter agency, and those induced by blood-poisoning with matter derived from without, it was not possible to draw the line. He believed that the cases of constitutional disturbance due to blood-poisoning were much more frequent than was generally supposed. The poison often worked itself off without producing many symptoms. It might, indeed, be that some one of the excretory organs was capable of ridding the organism of the poison at once. An illustration of one of the transient forms of infection of the blood, and the rapid recovery from it by elimination, is that which occurs in the dissecting room. Persons often experience a nausea or faintness, or notice a taste and smell which indicate the penetration into the system of volatile matter, the result of decomposition. This passes speedily away and is forgotten until some hours after, when the peculiar odor is again recognized in the perspiration, the excrement, or the breath.

The speaker would not give undue weight to the part blood poisoning plays in producing constitutional disturbance. The nervous system has its share. Thus the fever which results when the products of inflammation are bound down tightly by fascia, as in whitlow, or in abscess confined by bone, or in disease of a joint, must be provoked through the nervous spstem. The clinical features of the two forms of constitutional disturbance can be in most cases distinguished. In both these is usually pyrexia, with the rapid circulation and respiration, and the disorders of secretion usually accompanying fever. These signs were, as a rule, common to the two forms of mischief. But beyond these, in the more active forms of blood-poisoning, there are added rigors, sudden changes of temperature, profuse perspiration, etc. Often the distinction between these two forms of constitutional disturbance could not be made out in actual practice, because the two agencies, the nervous system and the blood, were both at work.

Following the address of the Chairman was a long and valuable discussion upon the "Treatment of Wounds." In this Mr. Lister took a large part. A discussion upon internal urethrotomy was opened by Sir Henry Thompson.

[An account of this will appear later in the RECORD.]

The address in the *Section of Obstetric Medicine* was delivered by W. S. Playfair, M.D., F.R.C.P., the subject being "The Teaching of Obstetric Medicine." The speaker described the deficiencies in obstetric medicine of the English schools. This branch of medicine, he said, though of high importance was made of but secondary importance in the regular lecture courses. The practical instruction was very small in amount, and the didactic lectures lasted only three months, or were relegated to the summer course. There had been many attempts to secure reform, but so far without success. Dr. Playfair thought that the General Medical Council ought to have among its members some physician who made a specialty of obstetrics and diseases of women.

Dr. Lombe Atthill introduced a discussion upon "Uterine Hæmostatics," with special reference to the hemorrhages occurring in the non-puerperal uterus. A large number of gentlemen took part in the debate, and the value of nearly every uterine hæmostatic was discussed more or less. Most of the speakers testified to the value of Chian turpentine in arresting hemorrhage and lessening pain, but it was thought oil of turpentine might act nearly as well. Hot water, ergotine (preferably injected into the gluteal muscles), injections of iron, plugging the cervical canal, pressure on the aorta, external and internal manipulation with the hands (in post-partum hemorage), the use of quinine, strychnine, and iron, as adjuvants, were all endorsed.

"Hemorrhage and sickness during pregnancy" was the title of a paper by Dr. Henry Bennett The speaker took the ground that chronic inflammation of the body of the uterus or of the cervix, very often existed in parturient women, and gave rise to many bad symptoms and accidents, such as laborious gestation, abortion, obstinate sickness, hemorrhage, etc. Dr. Bennett believed in treating these conditions, ulcerations of the neck, inflammations, etc., by surgical interference.

In the discussion Dr. Byrne, of Dublin, said that he did not believe in Dr. Bennett's views. He had seen very few cases of ulceration indeed.

Dr. Graily-Hewitt said that high authorities had stated that "inflammation" was a word which should be removed from uterine pathology.

A discussion on the treatment of uterine flexions was led off by Dr. Henry Gervis, and participated in by Drs. Herman Bantock, Pallen, Sims, and others. A paper was then read by Dr. Montrose A. Pallen, of New York, on the

"Etiology and Treatment of Lacerations on the Cervix Uteri." The paper, which strongly advocated the sewing up of all lacerations, and which pronounced lacerations to be of frequent occurrance, was evidently a novelty to the gynecologists present. It was discussed cautiously by several of the gentlemen. None except Dr. Hewitt, seemed to have ever seen many lacerations.

Mr. T. Spencer Wells opened a discussion on "the Removal of Uterine Tumors by Abdominal Section." He said that his whole experience in this operation included sixty cases; thirty-four of removal, with eighteen deaths and sixteen recoveries; twenty-six of incomplete operation, with one death. The mortality had been very much smaller since the introduction of antiseptic precautions. In describing the operation he referred to the importance of sewing up the uterine wound. When this was done, with antiseptic precautions, he thought the intraperitoneal method the better.

The discussion that followed was shared in by Mr. Knowsley Thornton, Mr. Lawson Tait, Dr. J. Marion Sims, Prof. Macleod, and others.

*The address in the Section of Psychology* was delivered by J. Crichton Browne, M.D. L.L.D., F.R.S.E., upon "Circles of Mental Disorder—Modern Nervous Diseases." The speaker began by deprecating the introduction of the subject of mental diseases into undergraduate education. He thought the curriculum already overburdened, and that the teachings of psychological medicine were now too ambiguous and unsystematic to be useful either in the matter of instruction or discipline. The student of medical psychology should first be broadly grounded on the principles of medicine.

The speaker then described the three divisions of mental disorders, which he made and which he represented by concentric circles. In the central circle were to be included all the insane and idiotic recognized as such. In Great Britian and Ireland, on January 1, 1880, there were 93,624 of these. Outside of this lies a second one which he would call the "crazy circle." In this we have those whose mental disease is only incipient or slight, such there as inoffensive lunatics, the host of eccentric, half-mad, crack-brained, and imbecile persons who move in every grade of society. Dr. Browne estimated that there were 180,000 occupants of the "crazy circle" in the United Kingdom. Finally, beyond the crazy circle lies another and outer circle, which might be called the neurotic. It embraced all the sufferers from nervous diseases that are not necessarily accompanied by mental disorder. No attempt was made to get a census of this class, but as 70,000 deaths are attributed annually to nervous diseases in England and Wales, it shows that the number of the neurotic circle is large.

The occupants of the three circles were constantly shifting from the inner to the outer, or the reverse ; sometimes slowly, sometimes rapidly. Neither the population nor the boundary limits of the circles were permanent and distinctly defined. The numbers, however of each are increasing. Dr. Browne gave his reasons briefly for believing that the number of the insane is increasing disproportionately to the population. He spent more time, however, in demonstrating the same thing for the circle of neurotics. He referred to Dr. Beard's conclusions regarding American nervousness, and thought that the same changes were taking place in England, though less noticeably than in America. His reasons for this belief in the increase of neurotic diseases were :

1. The fact that the English are a thinner people than they used to be. 2. That the death-rate from nervous diseases had not diminished, though many cases formerly classed under nervous diseases in mortality reports are not so assigned now, and though some nervous diseases tend to prolong rather than shorten life. 3. That certain diseases in which the nervous system plays a prominent part are on the increase, *e. g.*, diabetes, granular kidney, heart disease, aneurism, rheumatism, and gout. 4. Other points adduced were the increased frequency of premature baldness, decay of the teeth, the necessity which annual holidays have become, the rise of hospitals for nervous diseases, the rapid growth of neurological science, and the increased consumption of neurotic remedies.

The causes of this increase could be summed up under, 1st, The increasing complexity of the nervous system. 2d. The increasing complexity of life. Of the many conditions of modern life tending to promote the increase of mental and nervous diseases, Dr. Browne spoke especially of education, which, as now conducted, is full of danger to the young. The structural complexity of the brain was not reached at once nor at a single period of time. Different parts became perfected and capable of functioning at different times. The various powers of the mind ought to be cultivated then at the time that their structural basis was becoming developed. Certain mental capacities originally good might become utterly lost by lack of training. The speaker believed that muscular exercise, and especially manual dexterity, might help to develop mental qualities.

At the close of the President's address a discussion "on the influence of alcohol in the causation of insanity," was opened by Dr. G. W. Bacon, who was followed by Drs. H. Sutherland, Hack, Tuke and others. The general opinion seemed to be that the statistics on the point, so far given, greatly exaggerated the importance of the intemperance as a cause of insanity. It was often only an early symptom of the mental disease. Further and more exact data seemed to be needed. It was the opinion of Mr. Fletcher Beach that intemperance in parent was one of the prominent causes of imbecility in children.

Among a number of other papers presented was one by Lawson Taft, on a case of epileptic mania treated by oophorectomy. Considerable relief was brought about by the operation. In the discussion Dr. Bacon said that he castrated two male epileptics with the result in one case of great improvement.

*Section of Physiology.*—The session began with a discussion on "The Evidence derived from Clinical Observations and Physiological Experiments as to the Seat of the Formation of Urea in the Human Body," opened by Prof. Arthur Gamgee, of Manchester. After going over each point in the subject at length, he summed up his conclusions as follows: 1. It appears that the formation of urea does not occur, or occurs in insignificant amount (a) in the blood, (b) in the muscles, (c) in the nervous organs. 2. It is reasonable to suppose that in the glandular organs urea or its antecedents are formed. 3. The researches of various observers render it certain that urea is formed in the liver, though they do not warrant us in saying that the liver is the only seat of formation of urea. 4. Pathological observations establish a very strong presumption in favor of the liver being the organ in which the largest quantity of urea is formed. If properly pursued, pathological investigations afford the best possible means of proving conclusively whether urea is formed in the liver. 5. In order to obtain thoroughly satisfactory information from such investigations, it will be necessary that in future the total amount of nitrogen in the food shall be rigorously determined, as well as the total amount of nitrogen in the urine and the urea. Moreover, participation of other organs, and especially the kidneys, in the morbid condition supposed to be connected with a deficient formation of urea, must always be carefully investigatied.

A paper on "Urea in Blood and Muscle" was read by J. B. Haycraft, M.B. He found, on an average, in muscle, ten parts per 100,000; in blood, thirty parts. He believed urea to be a substance separated from the protein foodstuffs before they underwent tissue assimilation. It was, in fact, a phase of digestion.

Papers were read "On the Presence of Leucin and Tyrosin in the Urine in Numerous Diseases," on "The Action of the Ribs in Forced Expiration," on "The Contraction of Striated Muscle."

A lengthy discussion upon "Sleep and Hypnotism" was opened by Prof. W. Preyer, of Jena. The speaker described his own theory of the cause of sleep; that it was due to the accumulation of fatigue products in the circulation, which took up the oxygen of the blood that was needed by the nerve-cells. He then reviewed some of the experiments and studies in hypnotism made by Dr. James Braid, and recently by Heidenhain, himself and others. [A summary of these has appeared in the *Record*.

Dr. C. E. Brown-Sequard read a paper on the "Effect of

Certain Lesions of the Brain Upon the Excitability of the Motor Centres."

He related a number of experiments which showed that there were a number of lesions of the brain which could produce absolutely different effects. One of these is well known as motor inhibition (or paralysis); the other he called *dyamogeny.* As an example, he said that if a guinea-pig's head were suddenly crushed there would be paralysis of the fore legs, but very powerful convulsions in the hind legs. He had found that lesions near the base of the brain would produce like results in other animals.

*Section of Pathology.*—The event of this section was the address of the President, Sir James Paget, on "Elemental Pathology." The many difficulties surrounding the study of pathology, and the solution of its problems, were referred to. Some of these difficulties might be lessened and light thrown upon many pathological problems by the study of the "Diseases and Injuries of Plants." He then described some of the diseases which he himself had studied. Hypertrophies were important because they were not related to function but simply to supply of nutritive material; thus the "ringing" of trees causes an hypertrophy analogous to that produced in the human body by the obstructed return of lymph. Atrophies are rare in plants but degenerations are common. Senile degeneration is well illustrated by the changes which take place in leaves preceding their fall. The degenerations found in the "sere and yellow leaf" throw light upon symmetrical disease; they show that neither blood or nerves are necessary to symmetrical decay. Again, the mode of decay and fall of leaves show that decay is not death, for dead leaves do not fall; the necessary antecedent changes are vital processes. The low vitality, but high scale of development of the fibre of the leaf-stem, illustrated the fact that many changes which we call higher developments are, from the point of vitality, degenerations. Bone was allied to calcareous degeneration, though it had acquired high uses.

Regarding the repair of injuries to plants, he said it was much less perfect than in animals, the reparative process rarely proceeding beyond the mere protection of the idjured part. When plants are injured, layers of cork-cells are formed around, which may grow over the wound, or a gummy exhudation is poured out, but the epithelium never re-forms. Still, trees show "the inevitable process of repair" going on in one known case in a pine tree, for one hundred and fifty years without complete cure. This law, that an injury, which might occur at any age, and have no parallel antecedent in any ancestor, being indeed unique, should inevitably tend to become repaired in a particular way, indicated that there were other laws and properties at work in the body than those accounted for by heredity or evolution.

Two other topics were then taken up: inflammation and specific diseases. Inflammation was caused in plants by irritation. The gall was an illustration of the result of a specific inflammation, of which there were many kinds.

Sir James Paget concluded by urging his hearers to study some definite science apart from their professional work. "The astronomer studies astronomy in dark, stormy nights, and in the railway train, but his best studies are in the calm of the observatory and with a calm, clear sky. Our life-work lies in stormy nights and railway trains. If we desire to study calmly, and to profit, we must choose some subject which we can carry on apart from our professional work, during our leisure and holidays, which will yet afford lessons of surpassing value. Such a study is that of the diseases of plants

The address, says *The Lancet*, was characterized by all the ease and clearness of diction, masterly force of lauguage and felicity of illustration which distinguish the the speaker; and the audience showed no desire to break the spell which bound them in eager attention to the close.

Following the address, Prof. Joseph Lister read a paper of extreme interest upon "Micro-Organisms: Their Relation to Disease."

He first detailed the results of the experiments of Dr. Kock, of Woolstein. By ingenious methods of straining, that investigator had been able to detect not only the *bacil-*

*lus anthracis*, which causes anthrax, but he discovered other specific micro-organisms. These were the *bacillus septicæmiæ* and the chain micrococus, the latter always causing gangrene when injected into house mice.

The *bacillus septicæmiæ* is an extremely minute organism, and would be invisible unless stained. Dr. Koch had been able to prove that it always caused septicæmia, never pyæmia, when injected into mice.

Professor Lister then described the micro-organisms discovered by Toussaint in fowls suffering from fowl-cholera. This fowl-cholera is a highly infectious blood disease, characterized by swelling of the lymphatics of the neck, pericarditis, and duodenitis, but with no diarrhœa. The bacterium, claimed to be the cause of the fowl cholera, is oval shaped, multiplies by transverse construction, and is about 1-40000 of an inch in diameter. Pasteur has cultivated this organism, and has been able to modify it, so that by injecting the modified fluid he can cause a mild form of fowl cholera—protective against a second attack. In the same line of inquiry, Dr. Greenfield, by experiments, had recently shown that if a guinea pig be inoculated with the blood of a heifer suffering from splenic fever, the former animal takes the disease; if now the blood of the guinea pig be inoculated in a healthy heifer, it will protect that animal against splenic fever. Professor Toussaint had performed similar experiments in regard to "vaccinating" for anthrax. But he went farther; he showed that the prospective power is not conferred by inoculating any form of the *bacillus anthracis*, but by inoculating the fluid in which it had grown. Thus, he took blood from a case of anthrax, and by filtration and carbolic acid destroyed the organism; he then injected the filtrated fluid into a healthy animal, and found it protected against the anthrax.

In conclusion, the speaker described the ingenious experiments of Dr. Buchner, of Munich, by which he had transformed, by cultivation, the *bucillus anthracis* into the hay-bacillus, and *vice versa*.

At subsequent meetings of the Section, papers on "Glomerulo-Nephritis," by Dr. Leech, "The Pathology of

Psoriasis," by Dr. Thin, "Congenital Neurotic Papilloma," by Dr. Cotter, and "Cerebral Embolism," by Dr. Dickinson, were read.

Mr. Jonathan Hutchinson read a paper in which he reviewed the "errors which are liable to arise in the study of the part played by the nervous system in causing derangements of nutrition." Discussing the different views as to the action of nerves on nutrition, he divided them roughly into the view that all this action is vaso-motor, and to that which adds to this a special trophic influence. Before accepting the latter view, all hypotheses should be exhausted. He summed up the facts militating against the trophic hypothesis: the great rarity of some of the diseases which on the trophic theory should be common; the remarkable differences of these lesions supposed to be due to a common cause, and their great resemblance to lesions not of neurotic origin. Mr. Hutchinson gave a list of the conditions supposed to be dependent on trophic nervous influence. He then took up each separately and showed the doubts that might be thrown upon its neurotic origin.

In the discussion that followed, Drs. Brown-Sequard, Clifford Albutt, Dickinson and others took part, most of them disagreeing with Mr. Hutchinson.

Dr. Buzzard read a paper on "Some Cases of Joint Affection in Locomotor Ataxy" which was admirably illustrated by photographs.

*Section of Ophthalmology.*—This Section was opened with a few introductory remarks by its President, Mr. W. Bowman.

Mr. Priestley Smith then read a paper on the "Pathology of Glaucoma," which was subsequently discussed by Mr. Cowell, Professor Dunders and others.

In a sub-section of otology, several valuable papers were read. Among them was one upon "Electricity in Ear Diseases," and one upon the "Treatment of Suppuration of the Middle Ear." A discussion upon the comparative value of mechanical aids to hearing took place.

